# Quality Indicators but Not Admission Volumes of Neonatal Intensive Care Units Are Effective in Reducing Mortality Rates of Preterm Infants

**DOI:** 10.1371/journal.pone.0161030

**Published:** 2016-08-10

**Authors:** Niels Rochow, Erin Landau-Crangle, Sauyoung Lee, Holger Schünemann, Christoph Fusch

**Affiliations:** 1 Division of Neonatology, Department of Pediatrics, McMaster University, Hamilton, Canada; 2 Department of Clinical Epidemiology & Biostatistics, McMaster University, Hamilton, Canada; Hopital Robert Debre, FRANCE

## Abstract

**Aim:**

To investigate how two different strategies to form larger neonatal intensive care units (NICU) impact neonatal mortality rates.

**Methods:**

Cross-sectional study modeling admission volumes and mortality rates of 177,086 VLBW infants aggregated into 862 NICUs. Cumulative 3-year data was abstracted from Vermont Oxford Network. The model simulated a reduction in number of NICUs by stepwise exclusion using either admission volume (VOL) or quality (QUAL) cut-offs. After randomly redirecting infants of excluded to remaining NICUs resulting system mortality rates were calculated with and without adjusting for effects of experience levels (EL) using published data to reflect effects of different team-to-patient exposure.

**Results:**

The quality-based strategy is more effective in reducing mortality; while VOL alone was not able to reduce system mortality, QUAL already achieved a 5% improvement after reducing 8% of NICUs and redirecting 6% of infants. Including “EL”, a 5% improvement of mortality was achieved by reducing 77% (VOL) vs. 7% (QUAL) of NICUs and redirecting 54% (VOL) vs. 5% (QUAL) of VLBW infants, respectively.

**Conclusion:**

While a critical number of admissions is needed to maintain skills this study emphasizes the importance of including quality parameters to restructure neonatal care. The findings can be generalized to other medical fields.

## Introduction

The effect of admission volume-outcome relationship has been studied in adult medicine with the intention of improving quality of care and reducing costs. This topic is of ongoing interest in neonatal and pediatric health care research. The regionalization of perinatal centers by their annual admission volumes continues to be one of the most widely favoured approaches for reducing neonatal mortality rates and improving the quality of neonatal care and outcomes [1–9]. However, while the ultimate consequence of regionalization is to optimize the number of NICUs needed, it is of importance that appropriate units are being closed and that strategies are being put in place that the remaining units continue to improve their quality. Indeed, comparing mortality rates between various NICUs with different unit sizes has demonstrated an inverse trend of mortality rate decreasing with increased admission volume. Further, the effectiveness of this "volume-based referral initiative" approach has been shown to depend on the national organization of the units [2–6]. It was hypothesized that this improvement may be partially associated with the NICU team-to-patient exposure (experience level), where the neonatal outcome is influenced by the number of infants treated per physician and the frequency at which a physician performs certain procedures. Data from adult surgery demonstrated that greater admission volume and frequency of performing routine procedures correlate with better outcomes for various procedures, such as percutaneous coronary intervention, carotid endarterectomy, aortic-valve replacement, and some cancer resections [7]. The current explanation for the observed correlation is that teams in high-volume hospitals have increased exposure to specific diseases and procedures, and become more experienced in performing specific procedures than teams in low-volume hospitals, suggesting that high-volume hospitals achieve better outcomes [7, 10]. However, recent publications shed a different light on this conclusion. A closer look into published data showed that the variation of mortality rates between units of comparable sizes is significantly larger than between units of different sizes [4, 5, 11]. Using population based data, Shah et al. demonstrated in 2015 that smaller NICUs achieved a lower mortality rate when compared with larger NICUs [11]. A similar correlation was described recently in a paper by Horwitz et al. [12]. The authors found the lowest rates of unplanned adult readmissions in the lowest volume hospitals. In other words, the lowest volume hospitals had the best outcomes. The lower readmission rate could not be explained by hospital characteristics. Similar to NICU performance, there was also a wide variation of readmission rates between hospitals with similar sizes.

These observations cast doubt on the simplistic use of volume cutoffs for reducing VLBW infant mortality rates in NICUs [13]. It seems therefore to be reasonable to develop alternative quality-related approaches. The aim of this study is to test whether cutoff levels using either a volume- (VLBW infants admitted/year) or quality- (mortality rate/year) based strategy is best suited to reduce the system mortality rate.

## Methods

### Study design and sample

Cross sectional modeling study of VLBW infants weighing 501 to 1,500g admitted to hospitals with NICUs between Jan 1^st^ 2010 and Dec 31^st^ 2012 utilizing data provided by the Vermont Oxford Network (https://public.vtoxford.org, Burlington, Vermont) [14]. No hospital or patient identifiers were included, and no hospital or infant could be identified with the information given. The aggregated data included neonatal mortality rate and annual admission number of VLBW infants from 862 hospitals with 177,086 VLBW infants. For this study, only those hospitals that admitted at least 15 VLBW infants per year and provided a complete dataset during the three-year period from 2010 to 2012 were included, resulting in 729 hospitals with 166,718 VLBW infants.

The study was approved by the Research Ethics Board of McMaster University and the University of Vermont Institutional Review Board.

### Definition of modeling parameters

**Fig 1:**
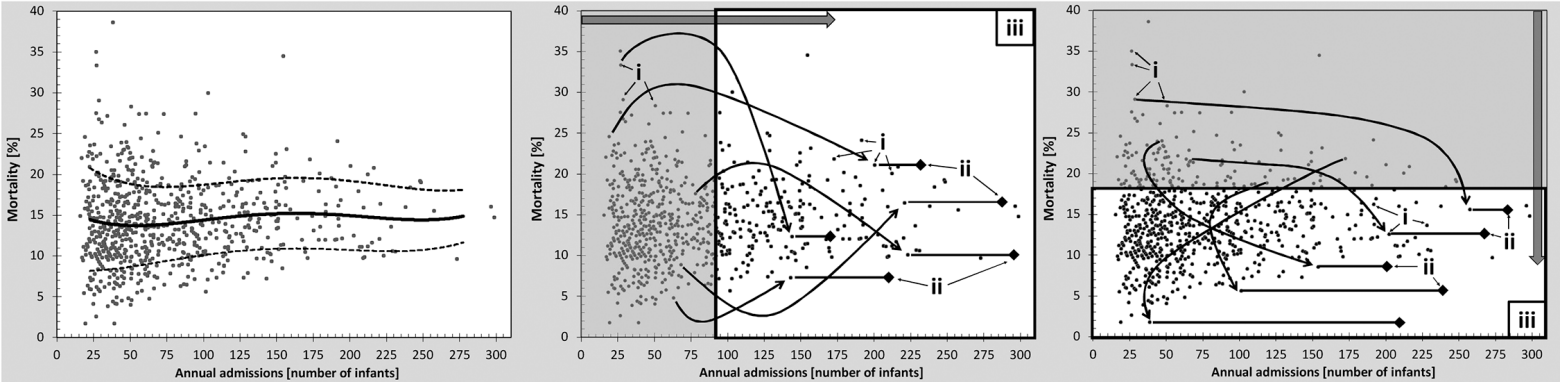
Stepwise random redirection of infants from hypothetically closed NICUs to remaining units, volume-based approach: cutoff is 95 VLBW infants admitted annually (left panel), quality-based approach: cutoff is 18% mortality rate (center panel), average mortality rate [%] by annual VLBW infant admissions of 729 hospitals with a total of 166,718 admissions during the three-year period from 2010 to 2012 (right panel). 1 –initial mortality, 2 –adapted mortality, 3 –system mortality; full line—mean; dotted line—standard deviation; shaded area shows units that will be closed.

The parameters used in this study are illustrated in Table 1 and Fig 1.

**Table 1:** Parameters used for modeling.

Variable	Description
Initial mortality rate	Raw mortality rate of a NICU as provided by VON dataset
Adapted mortality rate	Combined mortality rate of an individual NICU after receiving infants from a closed NICU
System mortality rate	Weighted average by number of admissions of adapted mortality rates for all remaining NICUs for a given cutoff value
Mean of system mortality rate	Average of system mortality rates using 1000 repetitions of random redirection for a given cutoff value
Average annual admission	Average number of admitted infants in the remaining units following the random redirection of infants for a given cutoff value

### Model design

Visual Basic for Microsoft^®^ Excel 2013 was used to program the algorithm for simulating the effect of volume–or quality–based regionalization of NICUs on mortality rate. Effectiveness of both strategies was analyzed, using the following five steps: (1) the units that did not meet given volume or quality cutoffs imposed by each respective strategy were virtually closed; (2) all infants of a closed unit were randomly redirected to one of the remaining units; (3) and were assumed to have acquired the mortality of the receiving unit (adapted mortality); (4) with all infants being redirected from closed units, the new system mortality was calculated as the sum of the products of the adapted mortality rate and total number of infants in each remaining unit after redirection divided by total number of infants in all units (Eq 1); (5) for each cutoff step, the random redirection was repeated 1000 times and the mean and confidence intervals of the resulting 1000 system mortality rates was calculated. The repetition generated a likelihood distribution of the new system mortality rates, which allowed the standard deviation to be determined for each cutoff (Fig 1).

$$system\;mortality=\frac{\sum \left(adapted\;mortality\;∙\;NICU\;admission\right)}{\sum NICU\;admission}$$ Eq 1

#### Volume-based strategy

The cutoff value for annual admission volume (x-axis) started at 16 infants/year and was increased by increments of 1 infant/year. Units that were below each cutoff were closed, and infants from each closed unit were randomly redirected to one of the remaining units (Fig 1).

#### Quality-based strategy

The steps to execute this strategy were identical to that of the volume-based strategy with the exception of the magnitude and scale of the cutoffs described above. The cutoff value started at a mortality rate (y-axis) of 38.5% and decreased after each step by an increment of 0.1% mortality. Units that were above the cutoff value were closed and infants from each of the closed units were randomly redirected to one of the remaining units (Fig 1).

#### Adjustments for effects of experience level

A relation between the annual admission volume and mortality rate of preterm infants is postulated. This is explained by higher training of medical staff for specific procedures and higher exposure to patients. For this effect the parameter of experience level will be defined. To account for this confounding element, the experience level was incorporated in an additional analysis. Data providing the relation between standardized mortality and annual admission volume was abstracted from recent publication [4]. Eq 2 represents the relation between the average standardized mortality and annual admission volume from abstracted data [4].

f(x)=−0.092∙ln(x)+1.43 Eq 2

where *x* is the annual admission volume of infants of a unit and *f*(*x*) is average standardized mortality rate. Eq 2 is included into Eq 3 for the calculation of the experience level-adapted mortality. Experience level-adapted mortality rate (*M*_*t*_*)* was quantified as the ratio of average standardized mortality rates post-redirection (*f(x*_*2*_*)*) to pre-redirection (*f(x*_*1*_*)*) multiplied by the adapted mortality rate (*M*_*a*_) of a given unit which receives infants from a closed unit.

$$M_{t}=M_{a}∙\frac{f(x_{2})}{f(x_{1})}$$ Eq 3

where *x*_1_ and *x*_2_ represent the initial and final number of infants in NICUs receiving infants from closed NICUs, respectively. The experience level-adapted mortality and annual admission volume of NICUs were used to calculate the experience level system mortality.

#### Comparison of the effects of combined volume and quality cutoffs on mortality rate

The combined effect of the quality and volume cutoff strategies on mortality rate was tested. This modeling first employed three quality cutoff levels that led to a 5%, 10%, or 15% improvement of the system mortality resulting in three new datasets. In a second step, the quality-based strategy was applied to these three datasets.

#### Validity of the model

As expected, the standard deviation (SD) of the likelihood function from 1000 repetitions increased with increasing number of unit closed, but was narrow for both volume- and quality-based strategies. The maximum value of 0.1% confirms the robustness of the model.

## Results

The study included 729 hospitals with 166,718 infants for final analysis. The baseline system mortality rate for all NICUs was 14.4% and mean annual admission volume was 76 ± 51 infants. Fig 1 shows individual mortality rates vs. annual admission volumes for all NICUs. Average mortality does not show significant variation over the full range of annual admission volumes. However, there is a wide scattering of mortality rates even between NICUs of comparable annual admission volume.

**Fig 2:**
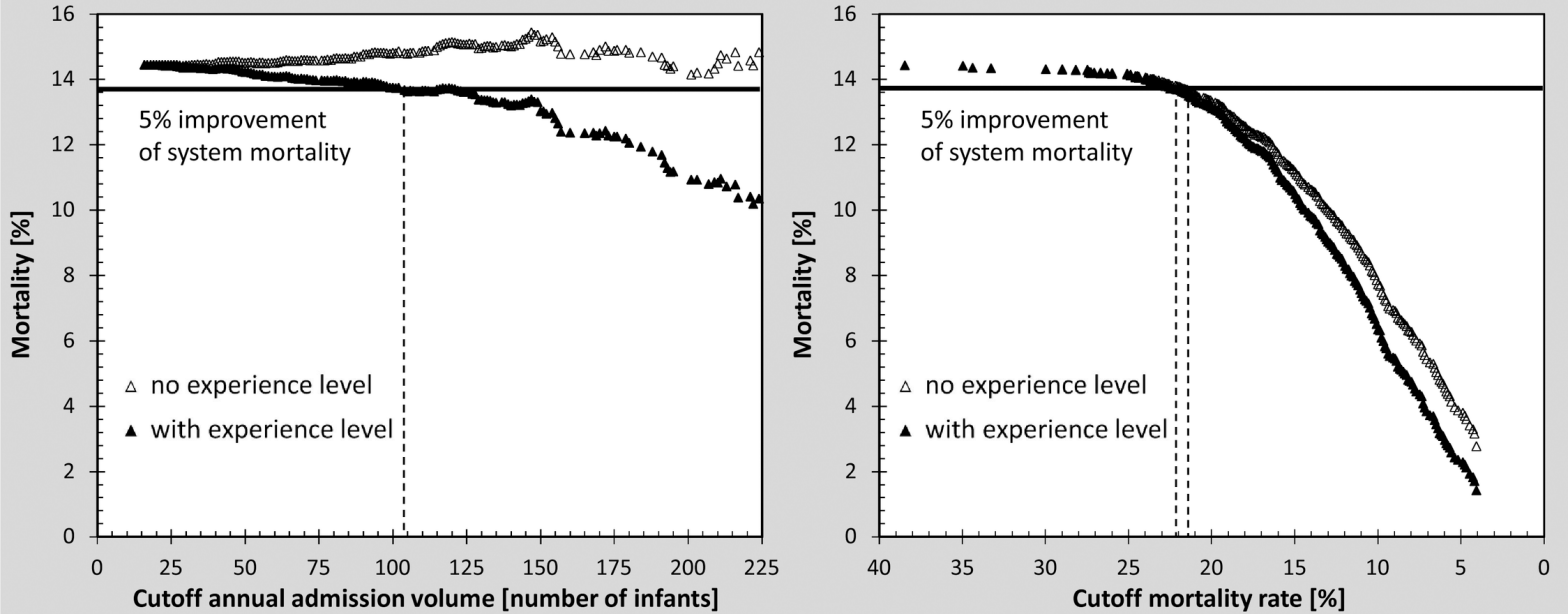
Comparison of cutoff effects on VLBW infant system mortality rate, with and without the effect of experience level, using a volume-based approach (left panel) and quality-based approach (right panel). The horizontal line indicates 5% improvement of system mortality.

**Fig 3:**
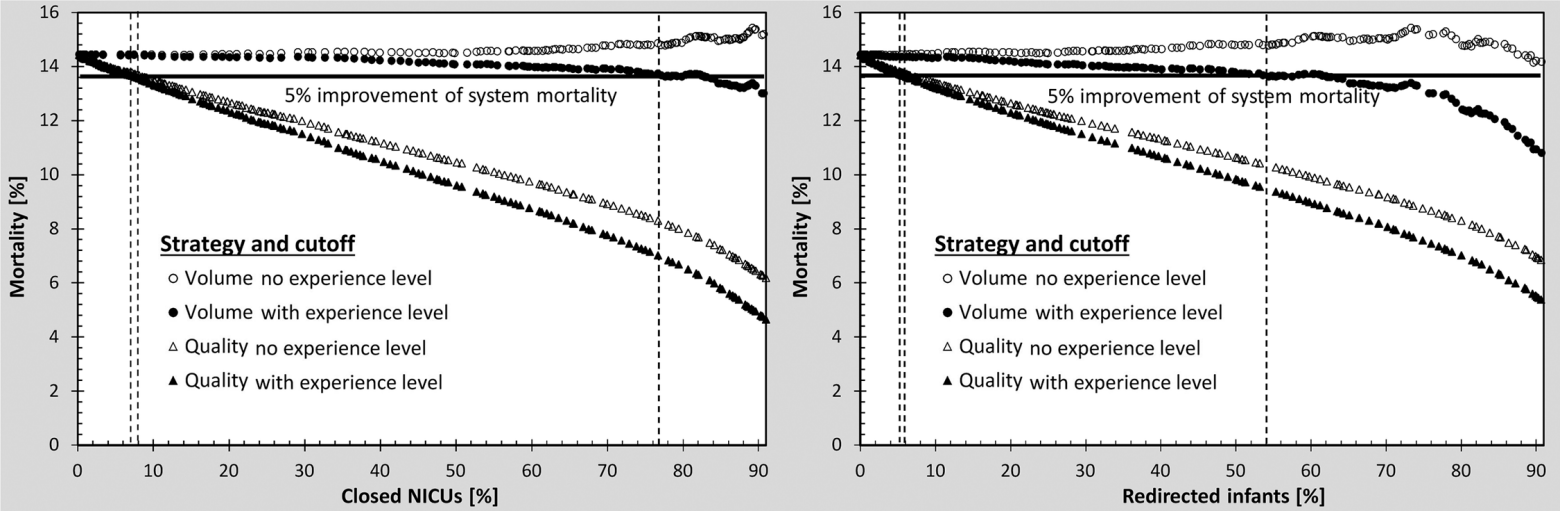
Comparison of VLBW infant system mortality rate when NICU closure is performed on volume-based and quality-based strategies. Mortality is presented for closed NICUs (left panel) and for redirected infants (right panel). The horizontal line indicates 5% improvement of system mortality.

Figs 2 and 3 show that quality-based strategies effectively improve the system mortality rate of VLBW infants. To achieve a 5% improvement in system mortality rate, a closure of only 8% of NICUs (Fig 3) accompanied with redirection of 6% of infants is required. Cutoffs based on volume do not improve system mortality rate. Unexpectedly, there is even a mild trend towards an increase of the system mortality rate towards higher cutoff levels for annual admission volume.

Figs 2 and 3 also show that the quality-based strategy further improved system mortality rates when experience level is incorporated into the model. To achieve a 5% improvement of the mortality rate, 7% of the NICUs need to be closed (Fig 3) with 5% of infants being redirected. With experience level in the model, the volume-based strategy also decreases the system mortality rate. However, compared to the quality-based strategy, approximately 11 times more hospitals (77% of NICUs) must be closed and infants redirected (54% of infants) to achieve the same improvement in system mortality rate. This translates into a cutoff level of average annual admission of 104 infants.

**Fig 4:**
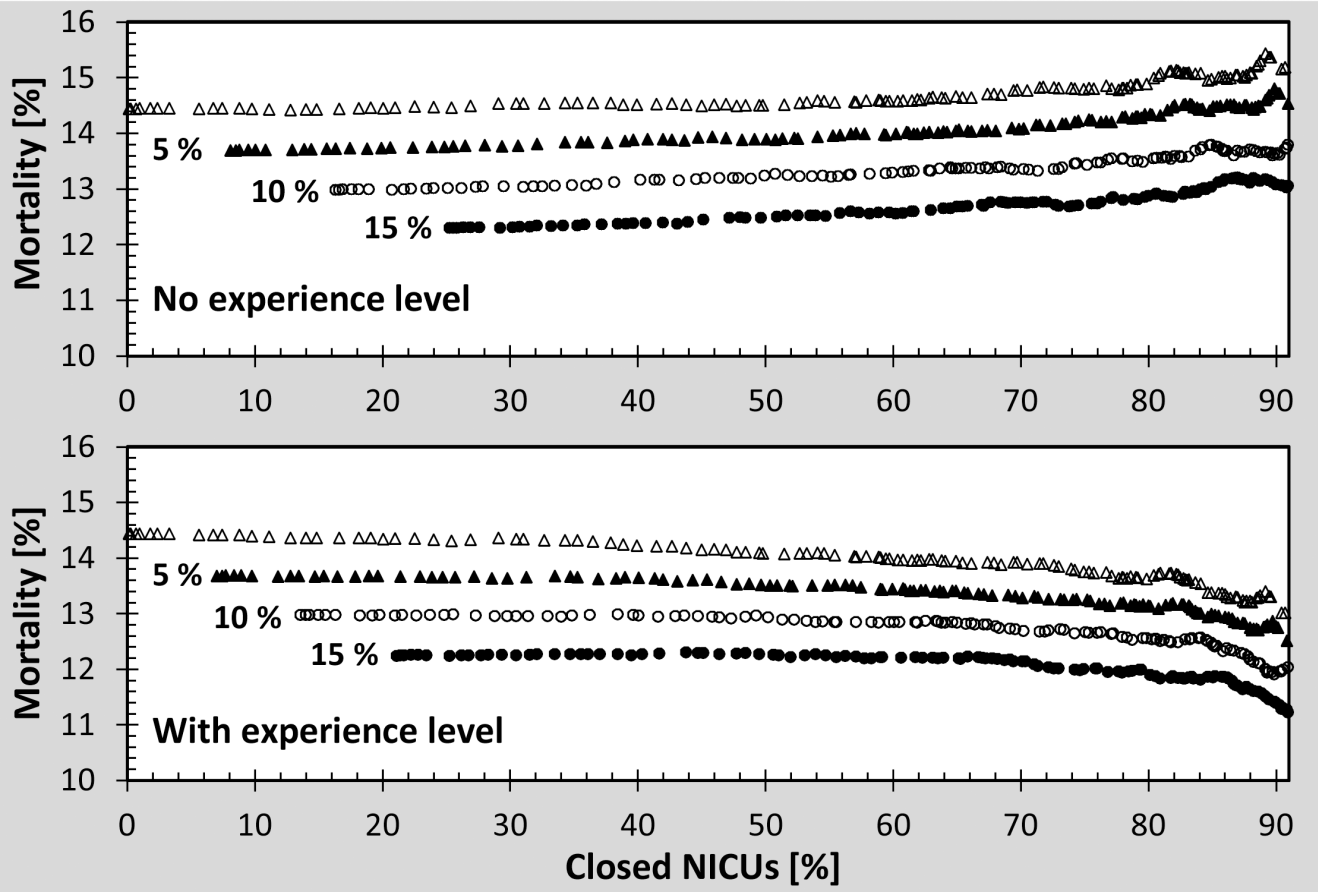
Combined mortality-based and volume-based strategy and its effect on mortality. Quality cutoffs 21.5%, 19.1%, and 17.2% were applied to the data, corresponding to 5%, 10% and 15% improvement in mortality rates, respectively. The mortality was plotted as a function of volume cutoffs without the effect of experience level (upper panel) and with the effect of the experience level (lower panel).

Fig 4 shows the combined impact of volume- and quality- based approaches with and without the experience level effect. The addition of volume-based strategies improves system mortality rate only when experience level is assumed.

## Discussion

In the present study we were able to show that neonatal outcomes can be improved by applying a quality-based rule for regionalization of NICUs. This approach is superior to one using only annual admission volumes. This superiority persists when the effect of experience level of the neonatal team is introduced in the model.

This study has several strengths: (i) The model uses real instead of hypothetical data to assess the effect of regionalization on mortality rate, and these results convincingly demonstrate how the strategies will perform when applied in a real-life scenario. (ii) The model includes a large number of patients and hospitals with a wide variation in outcome quality. It is also based on the average of the outcome data over three years. (iii) The Vermont-Oxford Network dataset was chosen as a representative data set because the Vermont Oxford Network provides a large sample size and high quality data. National and regional data are similar to this international data set, however usually of lower data density. (iv) The model does not make further assumptions. It closes NICUs, redirects all infants from one NICU to another, and calculates the new system mortality rate. It only requires annual number of admission and mortality rate of hospitals. In a secondary analysis, team-to-patient-exposure-level (TPEL) was added as an additional parameter to the model. The data for TPEL was derived from standardized mortality rates versus annual admission volumes of VLBW infants from a previous publication [4]. This incorporates the widely discussed assumption that forming larger NICUs will enhance the team function and skill level, and thus improve outcomes. (v) Further, the model is robust, as demonstrated by the small standard deviation of the system mortality rate at each cutoff for the two strategies. A precise estimate for mean and standard deviation of the mean of system mortality rate was achieved by repeating the random redirection of patients to remaining NICUs for each cutoff level 1000 times. Therefore, the presented model of regionalization can confidently be used for realistic inferences and comparison with previous studies.

Our study did not confirm that a volume-based strategy was able to significantly improve system mortality rates. A mild decrease was achieved only when the effect of team experience level was considered in the model. This failure is explained by the fact that variation of performance is by far, larger amongst NICUs of similar size than amongst NICUs with different sizes. In other words, the intra-class variation is higher than the inter-class variation. Following this observation, a major consequence could be to reduce the variation of performance between NICUs. Inevitably, relying on crude admission numbers bears the risk that smaller NICUs with excellent performance are being closed and patients will be redirected to larger units with potentially worse outcomes. It is obvious that such redirections will not enhance system performance nor reduce the intra-class variation of larger NICUs unaffected by the volume approach. In contrast, a strength of the quality-based approach is that it targets those NICUs with below-average quality of care in order to provide an opportunity for improvement. Interestingly, the quality approach will have an impact on the order with which NICUs would be targeted. Fig 1 shows that a significant number of low performance NICUs is found amongst low volume NICUs. Hence, the quality-based approach will first impact smaller NICUs similar to the volume-based approach. However, it will respect smaller units with high performance and will instead impact larger NICUs that perform below average.

Previous studies also could not identify a significant variation of mortality rates between centers of different sizes (small versus large) [3–5]. On average, larger NICUs do not show substantially lower mortality rates. A popular explanation commonly brought forward is that larger NICUs serve as referral centers that more frequently receive higher risk patients than smaller units with a lower mortality rate. However, this argument is not convincing as risk adjustment assessment for various hospitals demonstrated that on average there is no significant difference in the patient characteristics among units of different sizes [15]. This argument also does not explain the finding in this study that mortality rates vary by a factor of up to 3 amongst NICUs with similar annual admission volumes, regardless of whether they are referral centers or not. Such variation has been previously demonstrated in a number of studies [4, 13, 16, 17]. Rather than differences in patient characteristics, it is more likely that differences in the practice and structure of individual NICUs influences the mortality rate [18]. The existence of significant differences in NICU management on neonatal outcomes has already been classified 30 years ago and is also confirmed by more recent national and international benchmarking data [18, 19]. The key role of the NICU organization and management in reducing mortality rates has been identified by other studies [20]. For instance, it has been demonstrated that occupancy of NICUs has an effect on mortality rate. NICUs operating at lower occupancy had lower mortality rates compared to those at higher capacity [21]. The positive message of these findings is that units with higher mortality rates can improve their performance by adapting their practice. This has been shown for individual NICUs and for clusters of NICUs [19].

Another argument against a simple relationship of admission volume and mortality is that the number of patients that a neonatal team can responsibly take care of is limited. In relation to the outcome, there seems to be an optimal ratio of number of patients per team [11,20]. This might explain why the relationship between outcome quality and admission volume follows a U-shaped curve as previously shown [23]. Larger NICUs may be divided into subunits with fixed teams (microsystems). This suggests that the intensity of team-to-patient exposure determines the level of training and performance more than just the size of a unit. A recent study in adult intensive care also demonstrated a mirror-symmetrical relationship between mortality rates and the number of patients of the same diagnostic category [22, 23]. Mortality rates decreased with increased patient volume but after reaching a certain number of patients, the trend reversed and mortality rates increased with increased number of patients. In neonatal intensive care these findings seem to be equally plausible, and quite likely there is an optimum number of beds and patients a NICU team can successfully care for. This ideal workload per team seems to be in the range of approximately 100–120 VLBW admissions per year [4, 11, 20]. Once this range is exceeded, the performance of the NICU will decrease due to team overload. In light of this observation, a recent study from the Canadian Neonatal Network (CNN) is of great interest. The study showed that the outcome of smaller NICUs (<20 beds) was superior to those of larger NICUs (>20 beds) [11, 20].

Our model assumes that the hospitals in the Vermont-Oxford Network constitute a virtual health care system acting under one jurisdiction and similar economic constraints. A comparison of existing regional or national data sets demonstrate a similar distribution of mortality rates, especially with respect to its wide variation amongst units with similar sizes [3–5, 11, 13]. Therefore, this data set can be considered as an adequate surrogate to study these effects with a high data density and quality. Obviously, it would be of interest investigating regional or national differences in neonatal quality of care, but this is beyond the scope of this study.

In a real life scenario, when a NICU is closed patients are redirected to nearby NICUs with higher annual admission volumes or with lower mortality rates. In our data set, information about geographic location is not available. It is not believed that this limitation will have a major impact on the conclusions that the study draws. It could be speculated that variation in mortality rates arise from differences in health systems between regions, especially when using international data. A recent study confirmed quality differences between health systems of different regions. However, the effect size did not exceed a range of 10% compared to a 300% variation of outcome parameters observed in our sample [24]. Moreover, recently published quality data obtained from smaller healthcare networks of individual countries or states/provinces reveals that their intra- and inter-class variation of mortality rates and annual admission volumes are not different from our sample [5, 25]. These findings support two conclusions: (i) our sample is also representative for a smaller, more geographically-contained system and (ii) units that are geographically close to one another do not necessarily correlate in quality. These observations strengthen the validity of the results’ applicability to a single health system. As well, a potential systematic limitation such as geography would affect both the volume- and quality-based approaches in a similar way, thus should not impact the conclusion about the superiority of the quality-based approach to regionalization and improving quality of care. This is also accounted for in the 1000 repeated measurements of the model.

A limitation of the model is that mortality rates are not risk-adjusted. In particular, mortality rates at the earliest gestational ages may reflect differences in decision-making about infants of questionable viability rather than differences in quality. For example, if a family prefers to withhold aggressive life support and the infant dies this may be higher quality care than a prolonged and difficult NICU stay for that infant and family. However, the number of such extreme cases is quite small, especially given that our criteria include only infants with birth weights from 501g to 1,500g. This excludes the majority of infants that would be affected by the differential decision-making regarding the limits of viability. Given the available data, mortality rate is likely to be a feasible surrogate measure for quality of care.

Though our model takes data of health care quality provided for VLBW infants we feel that the results of this study are of general interest for health care research. First, our study affects a significant part of the population that is in need of highest quality of hospital care. Currently, about 7 to 10% of all newborns require specialized care after birth [26]. This number translates annually into 0.1 to 0.2% of the whole population. In our study, VLBW infants were analyzed because they reflect the bulk of the neonatal workload (not in terms of case numbers, but in terms of patient days) and because quality of care can be reliably measured in this patient group by contemporary benchmarking systems. We therefore hypothesized that mortality of those infants could be used as a surrogate for the overall quality of neonatal care. The other portion of the neonatal health care team’s workload—the late preterm and term infants—is also subjected to the specific unit’s performance. Data for late preterm and term infants are not collected by VON. In this context it is of interest to note that number of newborns receiving specialised care is comparable with annual incident rates of all major types of adult cancer [27].

We assume that the methodology used in our model can be equally expanded to other areas of medicine. Interestingly, similar to our results, Kuri et al. 2005 concluded using adult surgery outcome data that high-volume hospitals can deliver poor care and low-volume hospitals can deliver good care. Employing volume as a standard might not improve patient safety and may adversely affect the quality of surgical care [28]. Christian et al. 2005 reviewed approaches to study the volume–outcome relationship. The author concluded that understanding the underlying institutional factors that lead to the difference in outcomes is crucial. They further caution against oversimplified solutions, like hospital volume, that do not accurately address those factors [29].

In future studies, specific neonatal morbidities (ie. necrotizing enterocolitis, bronchopulmonary dysplasia, retinopathy of prematurity, intraventricular hemorrhage, sepsis) and composite measures using morbidity-free survival or scores such as Baby-MONITOR stratified by gestational age or birth weight should be analyzed [30]. Further, different scenarios could be considered by using data from national or regional benchmark systems and geographic locations in order to take into account travelling distances when re-directing patients from closed NICUs. Additionally, future studies could address the inter- and intra-country variation in NICU performance.

## Conclusions

Our study shows that a quality-based strategy has more power to improve the effectiveness of regionalized neonatal care than one based on annual admission figures.

Our data add thorough evidence that the annual admission volume of a unit is not a major determining factor of mortality rates and does not carry as much weight as previously claimed. It is of interest to note that these findings may also be generalized to those fields in adult medicine, where a minimum number of cases is needed to regulate access to specific diagnostics and therapies. As exemplified in the study by Horwitz et al, a higher volume of adult patients does not necessarily result in a higher performance [12].

In future studies this model can be further extended to apply other parameters of NICU quality such as morbidities of prematurity, to further assess how various regionalization strategies affect neonatal outcomes beyond mortality.
